# Population dynamics of potentially harmful haplotypes: a pedigree analysis

**DOI:** 10.1186/s12864-024-10407-x

**Published:** 2024-05-16

**Authors:** Katherine D. Arias, Iván Fernández, Juan Pablo Gutiérrez, Isabel Álvarez, Félix Goyache

**Affiliations:** 1 Área de Genética y Reproducción Animal, SERIDA-Deva, Camino de Rioseco 1225, Gijón, 33394 Spain; 2 Departamento de Producción Animal, Universidad Complutense de Madrid, Avda. Puerta de Hierro s/n, Madrid, 28040 Spain

**Keywords:** Linkage blocks, Population genomics, Pedigree variation, Potentially harmful alleles, Allele-Drop-In events

## Abstract

**Background:**

The identification of low-frequency haplotypes, never observed in homozygous state in a population, is considered informative on the presence of potentially harmful alleles (candidate alleles), putatively involved in inbreeding depression. Although identification of candidate alleles is challenging, studies analyzing the dynamics of potentially harmful alleles are lacking. A pedigree of the highly endangered Gochu Asturcelta pig breed, including 471 individuals belonging to 51 different families with at least 5 offspring each, was genotyped using the Axiom PigHDv1 Array (658,692 SNPs). Analyses were carried out on four different cohorts defined according to pedigree depth and at the whole population (WP) level.

**Results:**

The 4,470 Linkage Blocks (LB) identified in the Base Population (10 individuals), gathered a total of 16,981 alleles in the WP. Up to 5,466 (32%) haplotypes were statistically considered candidate alleles, 3,995 of them (73%) having one copy only. The number of alleles and candidate alleles varied across cohorts according to sample size. Up to 4,610 of the alleles identified in the WP (27% of the total) were present in one cohort only. Parentage analysis identified a total of 67,742 parent-offspring incompatibilities. The number of mismatches varied according to family size. Parent-offspring inconsistencies were identified in 98.2% of the candidate alleles and 100% of the LB in which they were located. Segregation analyses informed that most potential candidate alleles appeared *de novo* in the pedigree. Only 17 candidate alleles were identified in the boar, sow, and paternal and maternal grandparents and were considered segregants.

**Conclusions:**

Our results suggest that neither mutation nor recombination are the major forces causing the apparition of candidate alleles. Their occurrence is more likely caused by Allele-Drop-In events due to SNP calling errors. New alleles appear when wrongly called SNPs are used to construct haplotypes. The presence of candidate alleles in either parents or grandparents of the carrier individuals does not ensure that they are true alleles. Minimum Allele Frequency thresholds may remove informative alleles. Only fully segregant candidate alleles should be considered potentially harmful alleles. A set of 16 candidate genes, potentially involved in inbreeding depression, is described.

**Supplementary Information:**

The online version contains supplementary material available at 10.1186/s12864-024-10407-x.

## Background

Linkage disequilibrium (LD) may shape the genome causing its organization in block-like structures [1, 2], varying in size, usually called Linkage disequilibrium Blocks (LB). LB were defined as strong LD stretches flanked by genomic areas with higher recombination rates [3]. Although there exist different approaches to identify LB in non-related individuals [4, 5], LB show large variation both among populations and between individuals within population. LD patterns may vary due to genetic factors such as mutation and recombination but also to population events including selection, demographic changes, or drift [6, 7]. This is why, for practical applications, the ascertainment of patterns of haplotype conservation within LB is of primary interest.

Although LB are assumed to provide insights for disease association mapping and population genetics, there is an increasing interest in using them to explain the apparition of inbreeding depression in highly selected livestock populations [8, 9]. Since deleterious alleles are assumed to be fatal, low-frequency haplotypes, that are never observed in homozygous state in a population, are considered potentially lethal recessive genetic variants [8, 10–12]. However, the identification of low-frequency haplotypes requires a large number of genotyped individuals to ensure that they do not appear in homozygous state by chance [13, 14].

Detection of such genetic variants in large populations is difficult and subject to the occurrence of both false positives and false negatives [9]. Therefore, identification of potentially lethal haplotypes is usually carried out in two steps: first, their frequencies and their probabilities of being observed as homozygotes in a population are computed; and second, further restrictions such as their presence in one or both parents or grand-parents are applied [8, 9, 11]. The use of pedigree information may be even more important in populations with small to moderate sizes because lethal recessive variants have the potential to drift rapidly to higher frequencies due to inbreeding [15].

Although the potential role of mutation, demography, and selection on the frequency of deleterious alleles has been discussed before [7], little is known about the dynamics of potentially lethal haplotypes. Assuming that genetic variants causing inherited defects should expand in a population from founder events involving carriers [16], this research aims at the ascertainment of segregation patterns of haplotypes with low probabilities of being observed at a homozygous state using a small pedigree of the highly inbred Gochu Asturcelta pig breed.

The breeding history of Gochu Asturcelta pig can make the available pedigree informative for uncovering the dynamics of potentially lethal alleles. The highly endangered Gochu Asturcelta pig breed derives from four founders only [17, 18]. Wrong management practices, including full-sib matings, caused a sudden increase in inbreeding early after the start of the conservation programme [19] and the occurrence of inbreeding-related events such as stillborn parturitions that have been reported before in other inbred pig populations [20–22]. This scenario advised the implementation of a strict minimum coancestry mating policy [17] allowing, at present, a normal production and reproduction performance and, theoretically, the maintenance of lethal alleles in low frequency in the population and even purging.

In this research, carriers of potentially harmful haplotypes will be identified and the dynamics of such haplotypes, including Mendelian inheritance and parents-offspring inconsistencies, analyzed. Causes of deviation from the rules of Mendelian inheritance and the occurrence of alleles that are additional to the parental genotypes will be discussed. Segregating LB, carrying potentially lethal recessive haplotypes, will be subject to gene enrichment analyses to contribute to the knowledge of genomic areas potentially involved in inbreeding depression in pig.

## Methods

### Pedigree, cohorts and genotyping

A Gochu Asturcelta pig pedigree including 471 individuals (fully described in Supplementary Table S1), belonging to 51 different families (descendants of the same parental couple), was analyzed. This pedigree is a sample of that previously analyzed in Arias et al. (including 534 individuals, and 76 families) which was edited removing families with less than five offspring typed [23]. However, to gain consistency, pedigrees of two small families giving two boars (168 and 658) with good reproduction success were kept. Individuals were genotyped using the Axiom Porcine Genotyping Array (Axiom_PigHDv1; 658,692 SNPs). The typed individuals derived from 17 genotyped boars and 35 genotyped sows. Family size (number of offspring per parental couple) varied from 5 to 34, with 9 offspring on average. When necessary, the pedigree was visualized using the library visPedigree [24] of R environment.

For descriptive purposes, the individuals in the pedigree (whole population; WP) were split according to pedigree depth (equivalents to complete generations; *t*; [25]):

- Base population (BP) formed by 10 different individuals including: (i) two founders of the Gochu Asturcelta pig breed; (ii) four descendants of two untyped founders of the breed; and (iii) four direct descendants of the typed founders.
- G12 cohort, formed by 52 individuals with *t* ≤ 2 equivalents to complete generations and not included in the BP.
- G23 cohort, formed by 281 individuals with pedigree depth varying from *t* > 2 to *t* ≤ 3.
- G3 cohort, formed by 128 individuals with *t* > 3 in their pedigree.

The software Axiom Analysis Suite v4.0.3 (Thermo Fisher Scientific, Waltham, MA) was used to create standard .ped, .map, and .vcf files. SNPs were mapped using the *Sscrofa* genome build 11.1 [26]. Only autosomal chromosomes with known positions were considered. Following previous approaches, genotypes were only filtered on Mendelian errors to avoid the presence of null and false alleles [23, 27]. A total of 503,043 SNPs (372,402 of them polymorphic) with a minimum call rate of 0.97 were retained for further analysis. Missing genotypes were imputed using BEAGLE v5.4 [28, 29] using default parameters.

### Identification of LB and candidate haplotypes

Haplotype blocks (LB), defined following Gabriel et al. [3], were identified at a base population level using PLINK v1.9 [30] following the default procedure implemented in HAPLOVIEW v4.1 [1]. Since Veroneze et al. reported that the average extent of LD in pig was about 400 kb [31], the option *--blocks-max-kb* was fitted to 400. Considering that our dataset was previously filtered for Mendelian errors, no Minimum Allele Frequency thresholds were applied for the LB identification. Alleles (arbitrarily coded using three digits) and genotypes were identified per each LB, using our own R code and allelic frequency, *q*, computed.

Following Howard et al., potentially lethal recessive haplotypes were identified by computing haplotype frequency at both the population-wide and the within-cohort level [8]. The expected number of homozygotes (*E*[*H*]) will be calculated as *E*[*H*] = *q*^2^*N*, where *q* is the frequency of the haplotype and *N* is the total number of haplotyped individuals in each cohort (10, 52, 281, and 128, respectively) and the whole population (471). Assuming Poisson distribution, the probability of observing no homozygous haplotypes (*O*[*H*]) given *E*[*H*] as *P*(*O*[*H*] = 0|*E*[*H*]) = *e*^− *E*[*H*]^.

Haplotypes with *P*(*O*[*H*] = 0|*E*[*H*]) < 0.05 were considered candidate harmful haplotypes. Candidate haplotypes were identified at both the whole population level and the cohort level.

When necessary, relationships among candidate haplotypes were summarized with Venn diagrams constructed using the web-based software InteractiVenn [32].

### Parents-offspring inconsistencies

Haplotypes within LB were arbitrarily coded as codominant (microsatellite-like) marker alleles at the whole population level. The program COLONY 2.0.7.0 [33] was used to identify parent-offspring inconsistencies (i.e. departures of Mendelian inheritance). Analyses would inform on the consistency of the inheritance patterns across generations and families. The inconsistent haplotypes were identified using the genotypes inferred by the full-likelihood method [34] implemented in the COLONY and further assigned to the Offspring, the Father, or the Mother for each parent-offspring trio. The full-likelihood method [34] implemented in the COLONY pedigree uses the available pedigree, including family structures (known and excluded paternal and maternal sibships), and genotypes of related individuals. COLONY was run using the following settings: (a) polygamy with inbreeding as mating system; and (b) medium run lengths to search for the best assignment in the simulated annealing algorithm. Since parentage was previously verified using COLONY and SNP array data, the option *Known paternity* was fitted for all individuals in dataset.

Following Arias et al., parent-offspring incompatibility rates were quantified as: (a) Mean error rate per locus (*e*_*l*_) as *e*_*l*_ = *m*_*l*_/*nt* ; and (b) Mean error rate per allele (*e*_*a*_) computed as *e*_*a*_ = *m*_*a*_/2*nt*, were *m*_*l*_ is the number of single-locus genotypes including at least one allelic error, *m*_*a*_, the number of allelic mismatches, and *nt*, the number of replicated single-locus genotypes [27].

### Complementary analyses

Recombination rate (*ρ*) was estimated on phased genotypes using a machine-learning approach implemented in the R package FastEPRR [35]. Estimates were carried out on each porcine autosome with overlapping sliding windows under default settings for diploid organisms and window size fitted to 50 kb and step length fitted to 25 kb. To ascertain the relationships between LB and *ρ*, LB and *ρ* windows were overlapped using the *intersectBed* function of the BedTools software [36].

Furthermore, since genetic features such as Copy Number Variations (CNV) have been shown to cause calling errors giving wrong SNP alleles [27], the LB identified were overlapped, using BedTools as well, with 344 CNV regions previously identified in the Gochu Asturcelta pig population (see Supplementary Table S5 of Arias et al. [37]).

### Enrichment and functional annotation analyses

Following previous analyses [38], candidate haplotypes were subject to enrichment analyses using the BioMart tool [39]. Protein-coding genes found within these candidate haplotypes identified were retrieved from the Ensembl Genes 91 database, based on the *Sscrofa* v11.1 porcine reference genome. All the genes identified in the genomic areas spanned by candidate haplotypes were processed using the functional annotation tool implemented in DAVID Bioinformatics resources 6.8 [40] to determine enriched functional terms. An enrichment score of 1.3, which is equivalent to the Fisher exact test *P*-value of 0.05 [40], was used as a threshold to define the significantly enriched functional terms in comparison to the whole porcine reference genome background. Selection of significant composite annotation terms (clusters) using enrichment score as a criterion for selection rather than single annotation terms as independent statistically significant entities supports the identification of biological functions. In other words, this strategy allows to consider relationships between Gene Ontology annotation terms, by moving the analysis of biological function from the level of single genes to that of biological processes [40, 41].

## Results

### General overview

**Fig. 1:**
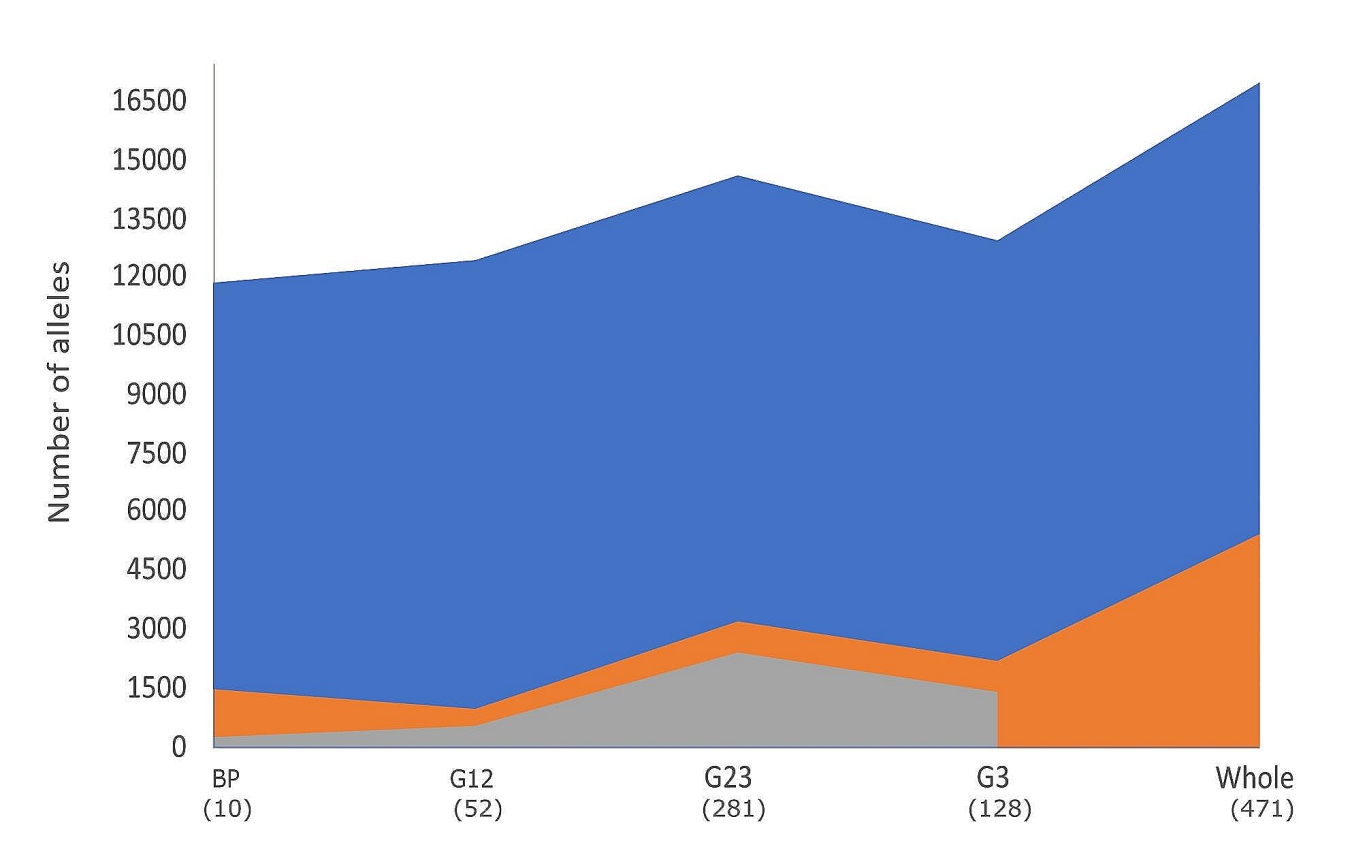
Allelic frequencies per cohort defined according to pedigree depth (BP, G12, G23 and G3), and in the whole typed population. Plot illustrates the total number of alleles identified (in blue), the number of candidate alleles (in orange), and the number of alleles that are identified in one cohort only (in grey). The number of individuals typed in each of the cohorts and in the whole population are in brackets

Up to 4,470 LB were identified in the BP, gathering a total of 16,981 alleles in the whole typed population (3.8 ± 2.9 alleles per LB, on average) and covering 236.2 Mb of the porcine genome (52.8 ± 93.9 kb on average; Table 1; Supplementary Table S2). Most LB (2,003; 45%) had two alleles only, 1,475 LB (33%) had more than four alleles, 232 (5%) had ten alleles or more, and 16 (0.4%) had 20 alleles or more. Furthermore, the number of alleles identified in each of the cohorts defined showed a large variation (Fig. 1). In the BP, the 4,470 LB had a total of 11,900 alleles. In the other cohorts the number of alleles increased with the number of individuals typed: 12,441 different alleles were identified in the 52 individuals forming cohort G12; 14,639 alleles were identified in the 281 individuals assigned to cohort G23; and 12,966 alleles were identified in the 128 individuals belonging to the cohort G3. The observed homozygosity in the whole population was 0.464.

**Table 1:** Number of Linkage Blocks (LB), mean length (in kb) and mean number of alleles per LB identified in the pedigree of Gochu Asturcelta analyzed per porcine chromosome. Minimum and maximum values are in brackets. Furthermore, mean recombination rate (*ρ* ± s.d.; in cM/Mb) and maximum Rho value (in brackets) are also given

SSC	LB	Length (kb)	Alleles	*ρ*
1	641	36.4 [0.9 ; 400]	3.1 [2 ; 18]	0.8 ± 2.4 [40.3]
2	388	45.4 [1.7 ; 400]	3.7 [2 ; 21]	2.2 ± 3.7 [36.3]
3	203	40.8 [0.9 ; 399.7]	3.3 [2 ; 14]	1.5 ± 2.2 [25.7]
4	300	47.3 [1.8 ; 400]	3.5 [2 ; 18]	3.4 ± 4.2 [28.7]
5	234	115.9 [2.8 ; 400]	4.8 [2 ; 17]	1.2 ± 2.1 [24.2]
6	277	36.5 [0.8 ; 399.9]	3.4 [2 ; 16]	1.1 ± 2.6 [36.8]
7	351	27.3 [2.7 ; 399.5]	3.0 [2 ; 12]	2.0 ± 3.6 [38]
8	174	39.0 [2.9 ; 399.5]	4.0 [2 ; 21]	2 ± 3.4 [33.9]
9	181	49.4 [3.6 ; 395.5]	4.3 [2 ; 20]	2.3 ± 4.4 [42.6]
10	251	35.0 [2.1 ; 400]	3.4 [2 ; 23]	4.4 ± 7 [62.7]
11	169	39.3 [3.8 ; 381.6]	4.2 [2 ; 24]	7.4 ± 6.2 [50.2]
12	143	89.9 [3 ; 400]	5.0 [2 ; 26]	1.8 ± 1.9 [9.7]
13	286	91.4 [2.0 ; 400]	4.7 [2 ; 31]	1.1 ± 2.9 [46.9]
14	165	75.7 [2.6 ; 399.9]	4.2 [2 ; 20]	1.3 ± 3.3 [89.7]
15	257	95.4 [2.6 ; 400]	4.8 [2 ; 19]	3.3 ± 3.8 [42.3]
16	205	30.6 [3.8 ; 341.9]	3.4 [2 ; 28]	2.8 ± 5.1 [75.2]
17	145	59.5 [2.5 ; 400]	4.5 [2 ; 21]	4.5 ± 5.2 [26]
18	100	38.9 [1.8 ; 297]	3.5 [2 ; 11]	3.6 ± 3.6 [39.6]
Totals	4,470	52.8 [0.8 ; 400]	3.8 [2 ; 31]	2.2 ± 3.9 [89.7]

The number of candidate alleles tended to vary across cohorts with the number of individuals typed as well (Fig. 1). Up to 1,516 (on 1,075 LB), 958 (on 694 LB), 3,230 (on 1,524 LB), and 2,193 (on 1,259 LB) alleles were considered candidate in the BP and cohorts G12, G23 and G3, respectively. Finally, 5,466 candidate alleles (32% of the total; gathering 8,587 copies) identified in the whole typed population, on 2,031 different LB (45% of the total), were considered candidates. Most candidate alleles identified at the whole population level (3,995; 73% of the total) had one copy only, 794 (15%) had two copies, 445 (8%) had three or four copies, and 232 (4%) from five (63) to nine (29) copies. Only three candidate alleles identified in the WP (two of them identified in the G23 cohort as well) had 1 homozygous genotype each (Supplementary Table S3).

**Fig. 2:**
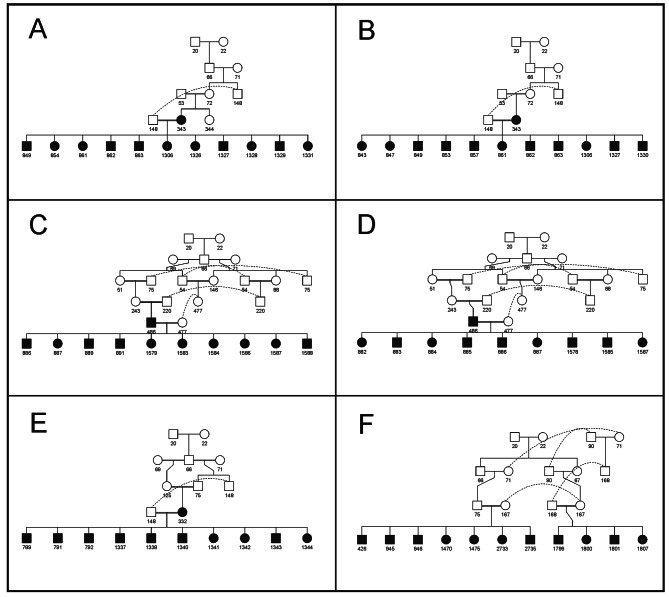
Pedigrees showing the segregation of six non-candidate alleles in the Gochu Asturcelta population analyzed. Boars are in squares and sows are in circles. Black squares and circles identify the carrier individuals. Plots A and B show the pedigree of sow 343 for alleles 104 on LB196 and 109 on LB1656, respectively. Plots C and D show the pedigree of boar 486 for alleles 112 on LB2925 and 109 on LB3315, respectively. Plot E shows the pedigree of sow 332 for allele 111 on LB645; and Plot F shows the pedigree of sow 167 (mated with two different boars) for allele 106 on LB3194. Only carrier offspring are included

Up to 4,610 of the alleles identified at the whole population level (27% of the total) were present in one cohort only (Fig. 1): 260 alleles were identified in the BP only and 528, 2,416, and 1,406 alleles were only identified within cohorts G12, G23, and G3, respectively. Of them, 181, 525, 2,410, and 1,399 were considered candidates within each cohort. Notably, six out of these 4,610 alleles (104 on LB196, 111 on LB645, 109 on LB1656, 112 on LB2925, 106 on LB3194, and 109 on LB3315) were not considered candidates at the WP level. Figure 2 illustrates the pedigrees of the carriers of these six non-candidate alleles. In five cases segregation involved one parent (sow 343 for alleles 104 on LB196 and 109 on LB1656; boar 486 for alleles 112 on LB2925 and 109 LB3315; and sow 332 for allele 111 on LB645) in which the alleles appear *de novo* and is transmitted to the offspring (from 9 to 11 descendants). Furthermore, one of these candidate alleles (allele 106 on LB3194; Fig. 2F) was identified *de novo* in 11 offspring of sow 167 (mated with two different boars) in which that allele was not present.

### Parent-offspring inconsistencies

A total of 67,742 parent-offspring incompatibilities (*e*_*a*_ = 0.016), involving 9,624 different alleles, were identified on 3,405 LB (*e*_*l*_ = 0.761) across the 471 individuals of the analyzed pedigree (Supplementary Table S4). Up to 5,229 parent-offspring incompatibilities involved the two alleles at a locus. A total of 63,016 parent-offspring incompatibilities were assigned to the offspring, 2,556 to the father, and 2,170 to the mother. The 3,405 LB on which paternal inconsistencies were identified were, on average, bigger (64.9 ± 104.4 kb vs. 14.1 ± 13.9 kb), gathered a higher number of alleles (4.3 ± 3.1 vs. 2.0 ± 0.3), and were less homozygous (observed homozygosity of 0.226 vs. 0.250) than the remnant 1,065 LB (Supplementary Table S2).

**Fig. 3:**
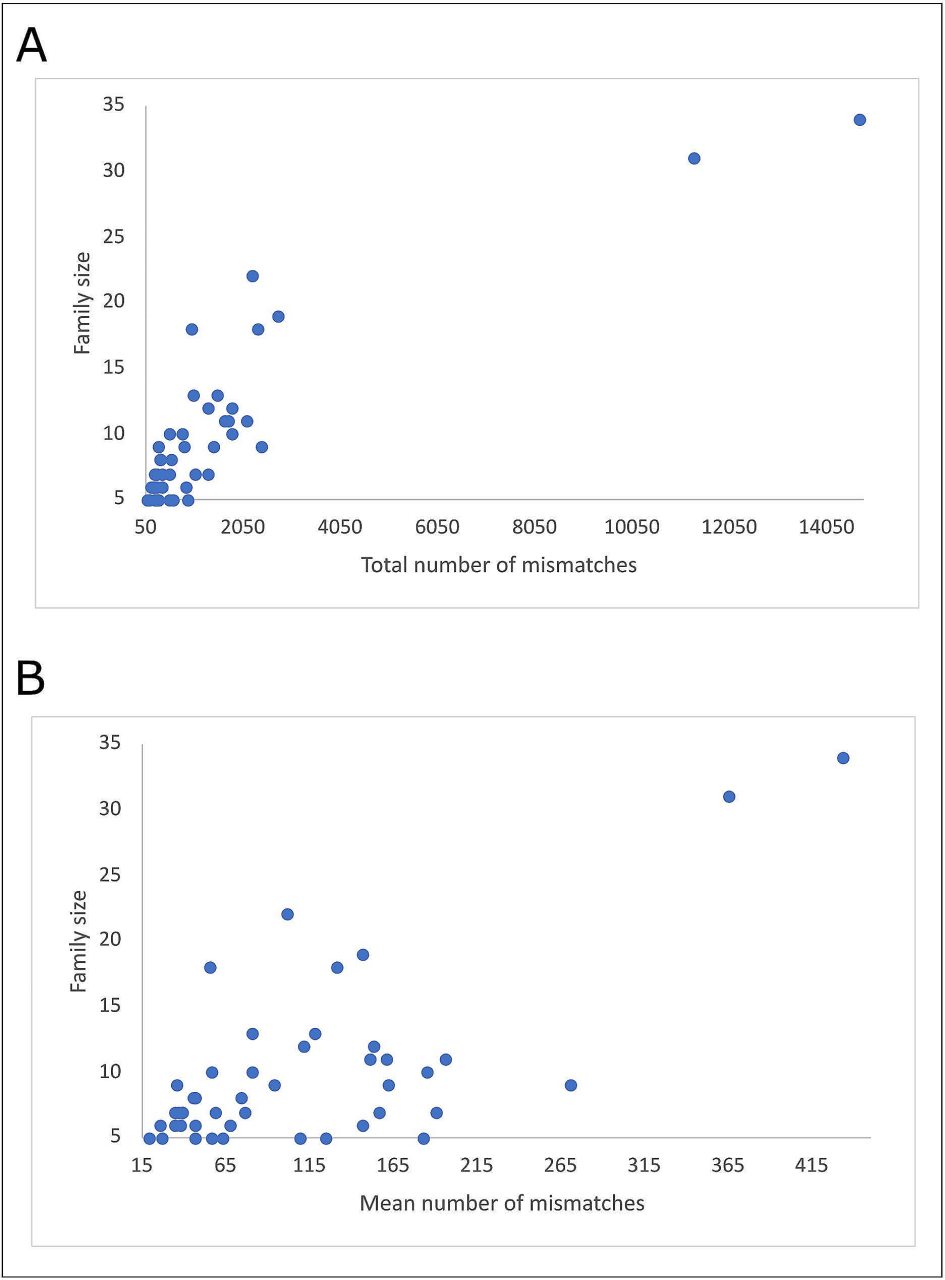
Dispersion plots constructed according to family size (on the Y-axis) and the total number of parents-offspring inconsistencies per family (on the X-axis; Plot A) and the mean number of parents-offspring inconsistencies per family (on the X-axis; Plot B)

Parents-offspring allelic incompatibilities varied across both cohorts and families according to sample size. In this respect, the BP gathered 1,009 errors (1.5% of the total), and cohorts G12, G23, and G3 gathered 6,609 (9.8%), 52,675 (77.8%) and 7,450 (11.0%) errors, respectively (Supplementary Table S4). Figure 3 illustrates how both total and mean number of mismatches per family varied according to family size. The two more-sized families (family 24 with 31 offspring and family 12 with 34 offspring) gathered a total of 11,326 and 14,742 mismatches, respectively.

**Fig. 4:**
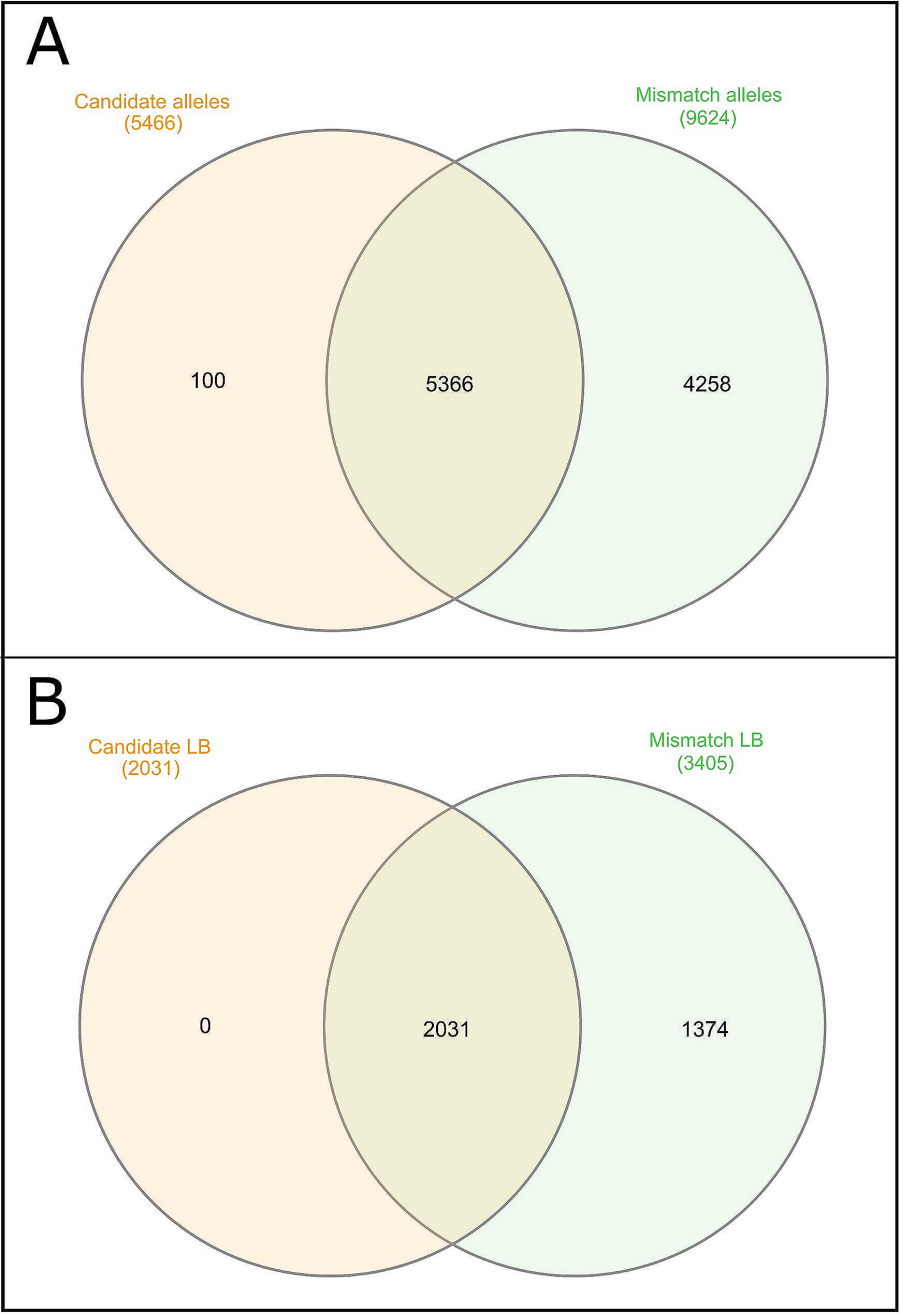
Venn diagrams summarizing the relationships between the 5,466 candidate alleles identified in the whole population and the 3,405 alleles involved in parents-offspring incompatibilities (Plot A). The relationships between the 3,405 LB on which parent-offspring allelic incompatibilities were identified and the 2,031 LB carrying candidate (potentially harmful) haplotypes at the whole population level are also summarized (Plot B)

Figure 4 illustrates the relationships between the 5,466 candidate alleles identified in the WP and their corresponding LB, and those in which parent-offspring inconsistencies were identified. Parent-offspring inconsistencies were identified in 98.2% of the candidate alleles and 100% of the corresponding LB.

### Segregation patterns of candidate alleles

**Fig. 5:**
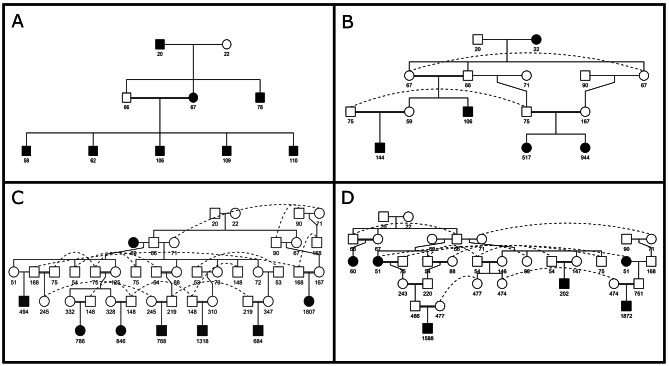
Pedigrees showing the segregation of four candidate alleles in the Gochu Asturcelta population analyzed. Boars are in squares and sows are in circles. Black squares and circles identify the carrier individuals. Plot A shows the fully segregant allele 101 on LB3529 which is transmitted from the founder boar 20 to the daughter 67 and five grandsons. Plots B (allele 102 on LB746), C (allele 105 on LB2481), and D (allele 103 on LB706) show pedigrees in which candidate alleles could be considered as identified *de novo* except for their identification in grandparents or great-grandparents

The segregation patterns of the candidate alleles showed considerable variation. Figure 5 contrasts a fully segregant candidate allele which is transmitted from the founder boar 20 to the daughter 67 and five grandsons (Plot A) with three pedigrees in which the identification of candidate alleles could be considered appearance *de novo* except for its identification, in some cases in grandparents or great-grandparents.

**Fig. 6:**
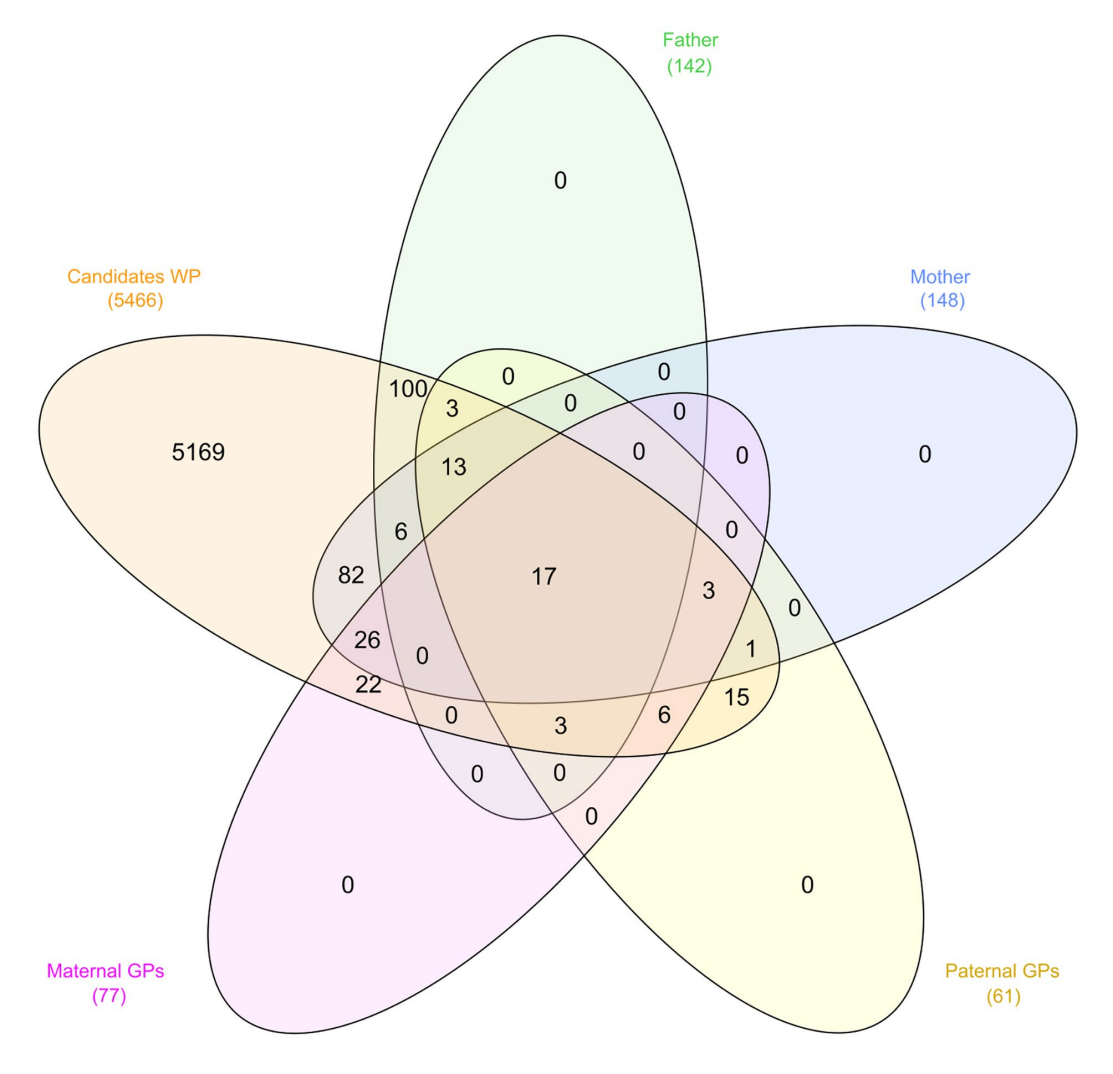
Venn diagram illustrating the frequency in which the 5,466 candidate alleles identified in the whole population could be identified in the fathers, the mothers, the paternal grandfathers, or the maternal grandfathers of the carrier

Figure 6 summarizes the frequency in which candidate alleles in the WP were identified in the fathers, the mothers, the paternal grandfathers, or the maternal grandfathers of the carrier individuals (see Supplementary Tables S2 and S3 as well). Up to 5,169 candidate alleles identified in the WP (94.6% of the total), gathering 6,774 copies (79% of the total copies of the candidate alleles), could not be identified in any ancestor of the carrier individuals.

A total of 188 alleles (3.4%), gathering 834 copies (1% of the total), were present in either the Father or the Mother of the carrier individuals but not in other ancestors. Moreover, 43 alleles (0.8%), gathering 138 copies (0.2%), were identified in at least one maternal or paternal Grand Parent of the carrier individuals but not in the parents (either sow or boar) of the carrier individuals.

**Fig. 7:**
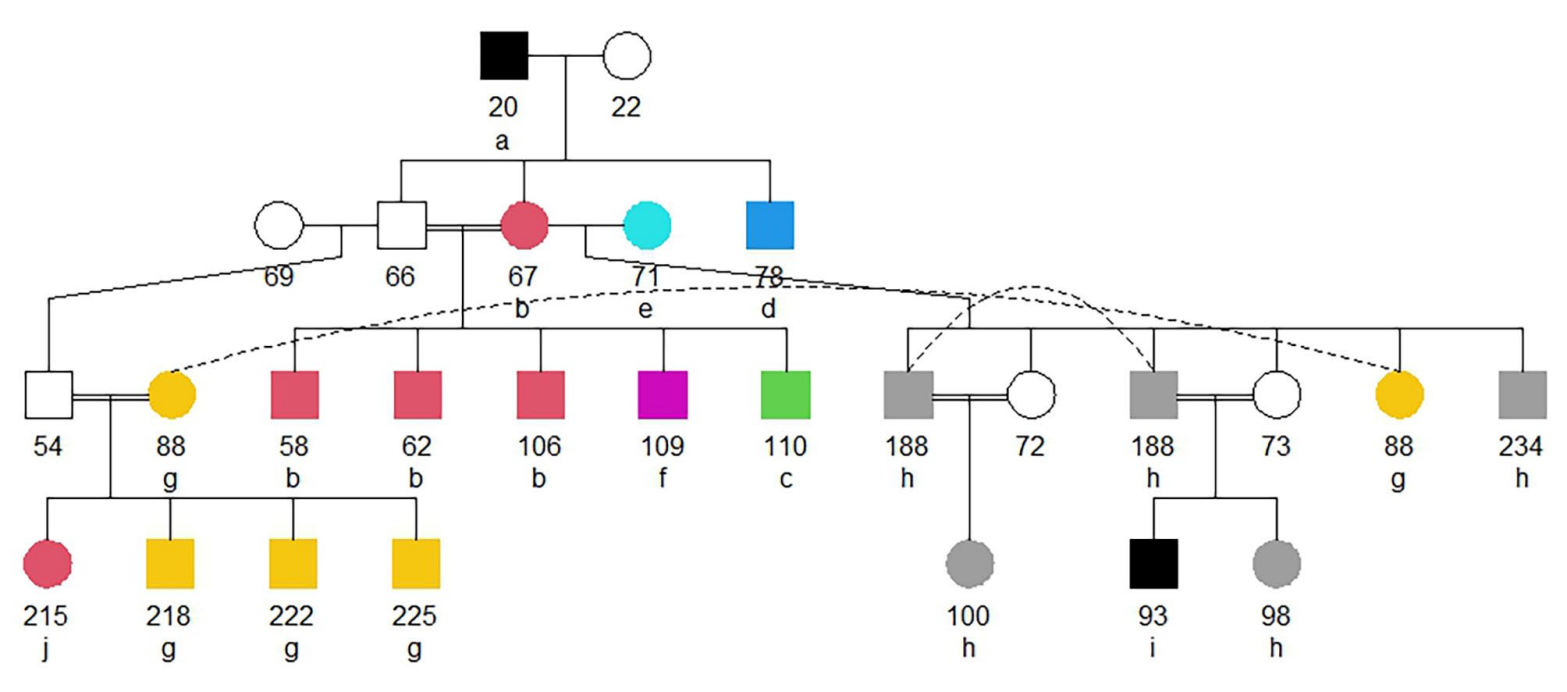
Pedigree illustrating the segregation of 17 segregant candidate alleles in the Gochu Asturcelta population analyzed. Boars are in squares and sows are in circles. Open squares and circles identify the non-carrier individuals. Letters beside the identification of the individuals mean that the individual carries segregant candidate haplotypes in the following LB (allele code in brackets): (A) LB1260 (101), LB1271 (101), LB3444 (103), LB3445 (106), LB3446 (106), LB3448 (104), LB3449 (104), LB3450 (103), LB3452 (104), LB3453 (103), LB3495 (101), LB3515 (101), LB3525 (101), LB3526 (101), LB3529 (101); (B) LB3444 (103), LB3445 (106), LB3446 (106), LB3448 (104), LB3449 (104), LB3450 (103), LB3452 (104), LB3453 (103), LB3495 (101), LB3515 (101), LB3525 (101), LB3526 (101), LB3529 (101); (C) LB3444 (103), LB3446 (106), LB3448 (104), LB3449 (104), LB3450 (103), LB3452 (104), LB3453 (103), LB3495 (101), LB3515 (101), LB3525 (101), LB3526 (101), LB3529 (101); (D) LB1260 (101), LB1271 (101), LB3495 (101), LB3515 (101), LB3525 (101), LB3526 (101), LB3529 (101); (E) LB1260 (101), LB1271 (101), LB1781 (103), LB1788 (103); (F) LB3525 (101), LB3526 (101), LB3529 (101); (G) LB1260 (101), LB1271 (101); (H) LB1781 (103), LB1788 (103); (I) LB1788 (103); and (J) LB1271 (101). Individuals carrying the same haplotypic combination are in the same color

Finally, a total of 66 candidate alleles (1.2%), gathering 470 copies (5.5%), were jointly identified in at least one parent (either sow or boar) and at least one maternal or paternal Grand Parent of the carrier individuals. Only 17 candidate alleles (0.3%), gathering 111 copies (1.3%), located on SSC4, SSC6, and SSC13, were identified in the boar, sow, and paternal and maternal grandparents and were, therefore, classified as segregating (Table 2). The LB on which these 17 segregating candidate alleles were identified lengthened 102.8 kb on average (min. 8.9 kb; max. 386.3 kb) and had 5.9 alleles on average (min. 3; max. 12). Figure 7 illustrates the variation of these 17 segregating candidate haplotypes. Interestingly, all of them were carried by founder individuals and were transmitted to progeny with pedigree depth up to 2.5 equivalents to complete generations. These 17 candidate alleles were selected for enrichment analyses.

**Table 2:** List of the 17 Linkage Blocks (LB) identified in the Gochu Asturcelta pig pedigree analyzed and selected for enrichment analyses. The porcine chromosome (SSC), position (in bp) of the start and end, the total Length (in bp), number of alleles (haplotypes), and the number of homozygous genotypes observed are given for each LB

LB	SSC	Start position (bp)	End position (bp)	Length (bp)	Alleles	Homozygous
LB1260	4	19,354,979	19,414,016	59,037	6	127
LB1271	4	22,309,905	22,377,024	67,119	4	160
LB1781	6	9,991,623	10,005,114	13,491	3	220
LB1788	6	12,126,135	12,135,042	8907	3	222
LB3444	13	138,485,747	138,569,542	83,795	6	229
LB3445	13	139,136,979	139,523,268	386,289	12	113
LB3446	13	141,327,231	141,490,185	162,954	10	115
LB3448	13	147,919,800	147,971,622	51,822	4	204
LB3449	13	148,026,119	148,049,808	23,689	4	238
LB3450	13	150,830,656	150,866,098	35,442	6	264
LB3452	13	151,058,135	151,193,312	135,177	7	179
LB3453	13	151,284,389	151,366,353	81,964	6	242
LB3495	13	169,746,824	169,879,566	132,742	7	265
LB3515	13	176,103,740	176,292,748	189,008	6	163
LB3525	13	179,090,359	179,263,134	172,775	9	231
LB3526	13	180,091,685	180,125,782	34,097	3	260
LB3529	13	180,324,134	180,433,044	108,910	5	210

### Relationships between LB, recombination rate and CNV regions

Mean *ρ* estimated for the whole typed population was 2.2 ± 3.9 4*N*_*e*_*r* (Table 1). To ascertain the possible influence of recombination in the occurrence of new alleles within LB, a total of 24,752 windows, with *ρ* higher than the mean, overlapped with the 4,470 LB identified. A total of 212 LB, gathering a total of 967 alleles (5.7% of the total number of alleles identified), overlapped with at least one window with *ρ* 2.5 s.d. above the mean, considered recombination hotspots (Supplementary Table S2). These 212 LB lengthened 53.8 ± 89.1 kb and gathered 4.6 ± 3.6 alleles, on average. The mean observed homozygosity in these 212 LB (0.453) was similar to that observed at the whole population level as well (Supplementary Table S2).

Furthermore, a total of 177 LB, gathering 804 different alleles (4.7% of the total number of alleles identified; 4.5 alleles per LB) overlapped with 72 different CNV regions (Supplementary Table S2) previously identified in Gochu Asturcelta pig [38]. These 177 LB lengthened 78.3 ± 115.2 kb on average and their mean number of alleles per locus was 4.7 ± 3.6. The observed homozygosity in these 177 LB was 0.457.

Only six different LB on SSC2, SSC6 and SSC11 (LB686, LB688, LB2023, LB2024, LB3086 and LB3087), gathering a total of 45 different alleles (7.5 alleles per LB), overlapped with both recombination hotspots and CNV regions. The observed homozygosity in these 177 LB was 0.459.

### Annotation and Enrichment analyses

A total of 16 protein coding-genes were located in the bounds of the 17 LB gathering the segregating candidate alleles, such as the syntaxin binding protein 5 L (*STXBP5L*) gene, the myosin heavy chain 15 (*MYH15*), the intraflagellar transport 57 (*IFT57*) gene, and the lipase I (*LIPI*) gene. A full description of these candidate genes, including their identification, description and location, retrieved from the Ensembl Genes 91 database, is given in Supplementary Table S5.

The genes identified formed two functional annotation clusters (AC; Supplementary Table S6), only one of them significant, putatively involved in immunity. The annotation cluster AC1 (enrichment score = 2.86; IPR007110:Immunoglobulin-like domain), included five candidate genes: CD86 molecule (*CD86*) gene, immunoglobulin like domain containing receptor 1 (*ILDR1*) gene, HERV-H LTR-associating 2 (*HHLA2*) gene, cell adhesion molecule 2 (*CADM2*) gene, and the roundabout guidance receptor 1 (*ROBO1*) gene. The non-significant annotation cluster AC2 (enrichment score = 0.32; AC2 GO:0016021 ∼ integral component of membrane) includes up to six candidate genes, four of them included in AC1 as well (*CD86*, *ILDR1*, *HHLA2* and *CADM2*), but also, the GABA type A receptor associated protein like 2 (*GABARAPL2*) gene and the ectonucleotide pyrophosphatase/phosphodiesterase 2 (*ENPP2*) gene.

## Discussion

The identification of potentially harmful alleles, by definition in very low frequency, is highly dependent on sample size. This is important because projects aiming at the identification of potentially harmful alleles require large sample sizes [8, 13, 42, 43]. Differences in sample size may partially explain the large differences in the identification of candidate haplotypes across cohorts and the WP in the current research. However, the close relationship between sample size and the number of potential candidate alleles in our data (Fig. 1), together with the high proportion of candidate alleles to the number of alleles identified (32% at the WP level), suggests that most candidate haplotypes identified are likely to be spurious.

Here, LB are identified at the BP, therefore making it possible to assume that most, if not all, allelic variants to be identified at the WP level should be present in the BP. However, our results substantially depart from that expectation. The number of alleles identified in the BP was 70% of those identified at the WP level only. Furthermore, it is intuitive to assume that only potentially harmful (candidate) allelic variants identified in the BP could be identified as candidate haplotypes in the WP. This is important because the identification of the same alleles in different generations is expected to give confidence on the allelic frequencies computed [11]. However, the number of alleles in low frequency probably being observed in heterozygous state only in the WP was 3.6-fold higher than those in the BP.

These facts illustrate a scenario in which the identification of haplotypes in very low frequency is subject to a high degree of uncertainty.

It is worth noting that our approach tried to avoid uncertainty as much as possible. Their identification at the BP gave a relatively low number of LB to be analyzed. When the presence of founders is not considered, the number of haplotypes substantially increases, virtually covering all analyzed genome adding uncertainty to the identification of potentially harmful haplotypes. When no pedigree was included in the analyses, PLINK identified roughly four-fold LB (16,793) in the whole typed population. Furthermore, the GHap R package [5] gave the identification of 20,681 different LB carrying a total of 308,497 alleles.

### Allele-Drop-in events

Events such as mutation or recombination may influence haplotypic frequencies [7]. In our case, however, since a significant number of alleles appear *de novo* in cohorts defined by their pedigree depth (Figs. 1, 2 and 5), we could discard that mutation causes the excess of variation identified. Although the role of recombination in the occurrence of new haplotypes within LB cannot be neglected, in our data the overlap of the LB identified with genomic areas potentially considered recombination hotspots is low and, therefore, recombination cannot explain by itself the excess of new alleles identified.

Although the alleles identified within LB fit well with the Mendelian expectations of inheritance, a non-negligible number of parent-offspring inconsistencies were identified. Both the candidate alleles and their LB coincided substantially with those in which paternity errors were identified (Figs. 4 and 6). Therefore, it is likely that causes of paternal incompatibility are related to those causing the occurrence of new alleles in low frequency at the WP level.

The paternal incompatibilities found are likely dependent on the occurrence of Allele-Drop-In (ADI; i.e. alleles that are additional to the parental genotypes) events. Figures 2 and 6 illustrate this. Validation of potentially harmful haplotypes usually relies upon their inheritance from genotyped ancestors [11]. However, in our data the identification of the candidate alleles in the parents and grandparents of the carrier individuals is extremely low (Fig. 6).

The analyzed dataset was edited to remove those SNPs in which Mendelian Errors were identified [23, 27]. In SNP arrays data, a relatively low number of Mendelian Errors identified in a very high number of loci are considered ADI events caused by the influence of genomic alterations, namely Copy Number Variations, SINEs, or LINEs [27]. Interestingly, consistently with the parental incompatibilities identified in the current research, Arias et al. reported that such Mendelian Errors increase with family size [27].

It is known that not all SNP genotyping errors depart from Mendelian rules of inheritance and, therefore, would not be identifiable. We suggest that such SNP calling errors causing ADI events are likely to be the main cause of the occurrence of ADI haplotypes. When constructing haplotypes in a LB, the presence of “hidden” calling errors at a locus, probably affecting a high number of loci in low frequency, being consistent with parental genotypes, causes the apparition of wrong alleles [27]. When used to construct haplotypes, the wrongly called SNP genotypes can cause the apparition of new variants. Since the wrongly called SNPs are in low frequency [27], ADI haplotypes are likely to appear in frequencies consistent with putatively harmful alleles, potentially darkening this kind of studies. Furthermore, since calling errors can be caused by genomic alterations such as Copy Number Variations, which can be inherited from parents to offspring [38], the calling errors could also be “inherited” in a relatively high frequency (Fig. 2). This may darkness the identification of potentially lethal alleles; their frequency may be relatively high if they are associated with heterozygous advantage due to positive pleiotropic effects.

The dense Gochu Asturcelta pedigree analyzed is relatively small. When the target is to identify haplotypes in very small frequency using populations including thousands of individuals, the number of “true” potentially harmful haplotypes will be extremely low if compared with those arising from ADI events, and their identification must be considered with caution.

This particularly applies to the criteria applied to remove false candidate alleles from data. In this respect, the separated assessment of the presence of the candidate haplotypes in either the parents or the (maternal and paternal) grandparents of the carrier individuals [8, 9, 11, 43, 44], although no doubt adding confidence to the correct identification of potentially harmful alleles, may not be enough. Some studies apply Minimum Allele Frequency (MAF) thresholds (e.g. MAF > 2%; [9] to remove “false” potentially harmful alleles from datasets. In our data, no candidate allele identified in the WP reached MAF = 1%, and MAF values for the 17 segregating candidate alleles identified varied from MAF = 0.53% (alleles 103 on LB1781 and 105 on LB3445) to MAF = 0.58% (alleles 101 on LB1271, LB3525, LB3526, and LB3529; Supplementary Table S3). In previous analyses using SNP arrays data, it has been suggested that the use of MAF thresholds may not be advisable if the goal is to keep in arrays data truly informative alleles [27]. In the case of haplotype construction, ADI alleles may easily exceed MAF thresholds, particularly if the analyzed population is large and includes big offspring size. ADI haplotypes may exceed MAF thresholds even if, as in our case, alleles considered candidates should never be observed as in homozygous state.

The assessment of the segregation of the potential candidate haplotypes identified at least two generations back from the carrier individuals can be a conservative approach to dealing with the identification of potentially lethal haplotypes.

### Putative role of candidate genes in inbreeding depression

Enrichment analyses were performed on the genomic areas of the LB in which segregating candidate alleles were identified to assess the possible role of the candidate genes identified in inbreeding depression. This is a difficult task because inbreeding depression effects are known to be heterogeneous along the genome, and the architecture of inbreeding depression is expected to differ between populations [45]. Causes of such differences may rely upon incomplete linkage disequilibrium between the causative lethal allele and the haplotypes inferred using marker information [9, 13, 14, 46, 47].

In any case, the 17 segregating candidate alleles identified fit well with expectations and the breeding history of the Gochu Asturcelta pig breed: they expanded in the population from founders [16], boar 20 and sow 71, and could have been purged from the population after the implementation of strict mating policies in the conservation programme [17].

A significant part of the candidate genes identified, such as the T-cell antigen receptor *CD86* gene, involved in nutrient absorption and gut barrier function in pigs [48], *ILDR2*, involved in the formation of epithelial cells barrier [49], *HHLA2*, belonging to the B7 gene family can substantially regulate T-cell activity [50], *GABARAPL2* involved in macroautophagy which plays essential roles during development and immunological phenomena [51], the *ABCC13* gene, involved in xenobiotic defense [52], or the *IFT57* gene, involved in inflammatory responses often associated with elevated cytokine concentrations [53], are expected to participate defense and immune response. However, it has been suggested that genes associated to immunity can have a pleiotropic effect [54]. Furthermore, it is worth mentioning that the *GABARAPL2* gene can be involved in wound healing [55, 56], the *HHLA2* gene is involved in sow fecundity and litter size by interacting with peri-implantation endometrium in Yorkshire pigs [57] and that the *IFT57* gene has been associated with the regulation of the embryonic development causing short stature and brachymesophalangia in humans [58]. Although pleiotropy could be the rule in the mammal genome [44, 59] and, therefore, it is always possible to find consistency between any list of candidate genes obtained at random and a given trait under study, the candidate genes located in the bounds of the haplotypes considered candidate in this study would fit well with expectations of possible effects of potentially harmful alleles on inbreeding depression.

## Conclusions

The population dynamics of potentially harmful haplotypes suggest that most alleles identified in very low frequencies in a pedigree can be considered Allele-Drop-In events. The number of candidate alleles probably never identified in homozygous state in the pedigree analyzed substantially exceeded the expectations and it cannot be explained by mutation or recombination. The *de novo* occurrence of haplotypes in a LB is likely to be related to SNP calling errors. In cases of dense pedigrees, such calling errors can cause that these *de novo* haplotypes identified in a parent or grandparent being erroneously identified in the offspring or grand-offspring as well despite its lack of biological meaning. The number of segregating haplotypes (being identifiable at least in both the parents and the grandparents of the carrier individuals) is very low, and it would be advisable to restrict the identification of potentially harmful haplotypes to that scenario. Furthermore, the use of haplotypic segregation can be a promising strategy to identify calling errors in SNP genotyping.

### Electronic supplementary material

Below is the link to the electronic supplementary material.

Supplementary Material 1

## Data Availability

All genotypic data (4,470 LB and 471 individuals) necessary to replicate the results presented are in a plain text file (16.5 MB) that can be downloaded from https://drive.google.com/file/d/1NYKNsuk6IqutnpCCLpUjDl6O7CQL6Gx6/. SNP array data are under currently analysis within the “AutoGenome” project framework. However, the data set used is available from the corresponding author upon reasonable request.

## Abbreviations

BP: Base population
4*N*_*e*_*r*: Recombination units, where *N*_*e*_ is the effective population size and *r* the recombination rate for the window
G12: Cohort formed by individuals with *t* ≤ 2
G23: Cohort formed by individuals with pedigree depth varying from *t* > 2 to *t* ≤ 3
G3: Cohort formed by individuals with *t* > 3
LB: Linkage disequilibrium Blocks
LD: Linkage disequilibrium
*t*: Equivalents to complete generations
WP: Whole population
